# Synergistic Imaging: Combined Lung Ultrasound and Low-Dose Chest CT for Quantitative Assessment of COVID-19 Severity—A Prospective Observational Study

**DOI:** 10.3390/diagnostics15151875

**Published:** 2025-07-26

**Authors:** Andrzej Górecki, Piotr Piech, Karolina Kołodziejczyk, Ada Jankowska, Zuzanna Szostak, Anna Bronikowska, Bartosz Borowski, Grzegorz Staśkiewicz

**Affiliations:** 1 Medical Diagnostic Center Voxel, Regional Hospital in Łańcut, Ignacego Paderewskiego 5, 37-100 Łańcut, Poland; 2 Department of Correct, Clinical and Imaging Anatomy, Medical University of Lublin, Chodźki 4 (CSM), 20-093 Lublin, Poland; 3 Department of Orthopedics and Traumatology, Medical University, Jaczewskiego 8, 20-090 Lublin, Poland; 4 Department of Radiology, Medical University of Lublin, Jaczewskiego 8, 20-090 Lublin, Poland

**Keywords:** COVID-19, lung ultrasound, low-dose computed tomography, chest CT severity score (CTSS), lung ultrasound severity score (LUSS), pulmonary inflammation, disease severity assessment

## Abstract

**Background/Objectives:** To assess quantitatively the correlation between the lung ultrasound severity scores (LUSSs) and chest CT severity scores (CTSSs) derived from low-dose computed tomography (LDCT) for evaluating pulmonary inflammation in COVID-19 patients. **Methods:** In this prospective observational study, from an initial cohort of 1000 patients, 555 adults (≥18 years) with confirmed COVID-19 were enrolled based on inclusion criteria. All underwent LDCT imaging, scored by the CTSS (0–25 points), quantifying involvement across five lung lobes. Lung ultrasound examinations using standardized semi-quantitative scales for the B-line (LUSS B) and consolidation (LUSS C) were performed in a subgroup of 170 patients; 110 had follow-up imaging after one week. Correlation analyses included Spearman’s and Pearson’s coefficients. **Results:** Significant positive correlations were found between the CTSS and both the LUSS B (r = 0.32; *p* < 0.001) and LUSS C (r = 0.24; *p* = 0.006), with the LUSS B showing a slightly stronger relationship. Each incremental increase in the LUSS B corresponded to an average increase of 0.18 CTSS points, whereas a one-point increase in the LUSS C corresponded to a 0.27-point CTSS increase. The mean influence of the LUSS on CTSS was 8.0%. Neither ultrasound score significantly predicted ICU admission or mortality (*p* > 0.05). **Conclusion:** Standardized lung ultrasound severity scores show a significant correlation with low-dose CT in assessing pulmonary involvement in COVID-19, particularly for the B-line artifacts. Lung ultrasound represents a valuable bedside tool, complementing—but not substituting—CT in predicting clinical severity. Integrating both imaging modalities may enable the acquisition of complementary bedside information and facilitate dynamic monitoring of disease progression.

## 1. Introduction

The outbreak of the COVID-19 pandemic, caused by the SARS-CoV-2 virus, has emerged as one of the most severe global health crises of the 21st century. The disease rapidly spread worldwide, leading to millions of infections and placing immense strain on healthcare systems [1]. Given the dynamic course of the infection, the heterogeneous clinical presentation, and limited availability of molecular tests, imaging modalities have assumed a pivotal role in the diagnosis and monitoring of COVID-19. Among these, low-dose computed tomography (LDCT) has been recognized as one of the most sensitive tools for detecting inflammatory pulmonary lesions, allowing precise evaluation of their extent and anatomical distribution [2]. Although reverse transcription polymerase chain reaction (RT-PCR) testing remains the diagnostic gold standard for COVID-19, LDCT has gained prominence due to its high diagnostic accuracy. Nonetheless, its routine clinical application involves radiation exposure and limited equipment availability, particularly in overwhelmed hospital settings [3]. Concurrent with the growing importance of LDCT, lung ultrasound (LUS) has progressively gained attention, providing rapid, repeatable, and safe bedside imaging without the necessity of patient transport. Despite their differing technical foundations, these two imaging methods have proven complementary: LDCT offers superior diagnostic precision, whereas LUS excels in mobility and safety. In resource-constrained situations, including limited intensive care unit beds or restricted oxygen therapy availability, accurate assessment of lung involvement using imaging techniques may critically influence therapeutic decision-making [4].

Notably, the contemporary approach to COVID-19 imaging transcends mere diagnostic aims, increasingly focusing on predicting disease severity and identifying patients requiring intensive monitoring. Within this framework, standardized assessment scales such as the lung ultrasound severity score (LUSS) and chest CT severity score (CTSS), enabling the semi-quantitative evaluation of inflammatory changes, have become increasingly relevant, aiding clinical prognostication. However, clearly defined algorithms specifying the precise role and integration of these imaging modalities in managing COVID-19 patients remain elusive. Although previous studies have demonstrated correlations between the LUSSs and CTSSs, many were limited by small sample sizes, heterogeneous ultrasound protocols, and a lack of quantitative analysis. Therefore, our study aimed to address these gaps by employing a large cohort assessed with a standardized LUS protocol to enhance the reproducibility and generalizability of findings. Addressing these challenges necessitates innovative technological approaches, including artificial intelligence (AI), which could enhance the development of advanced diagnostic methods, supporting healthcare systems in infection identification and surveillance. Therefore, this study aims to quantitatively evaluate the correlation between the lung ultrasound and low-dose computed tomography in predicting COVID-19 disease progression, supported by artificial intelligence methods. It was hypothesized that the LUSS B would demonstrate a stronger correlation with the CTSS than the LUSS C, thereby indicating superior sensitivity for detecting pulmonary involvement in patients with COVID-19. Ultimately, this research seeks to provide a foundation for optimizing imaging strategies in patients with a suspected or confirmed SARS-CoV-2 infection, considering both diagnostic effectiveness and patient safety.

## 2. Materials and Methods

**Figure 1:**
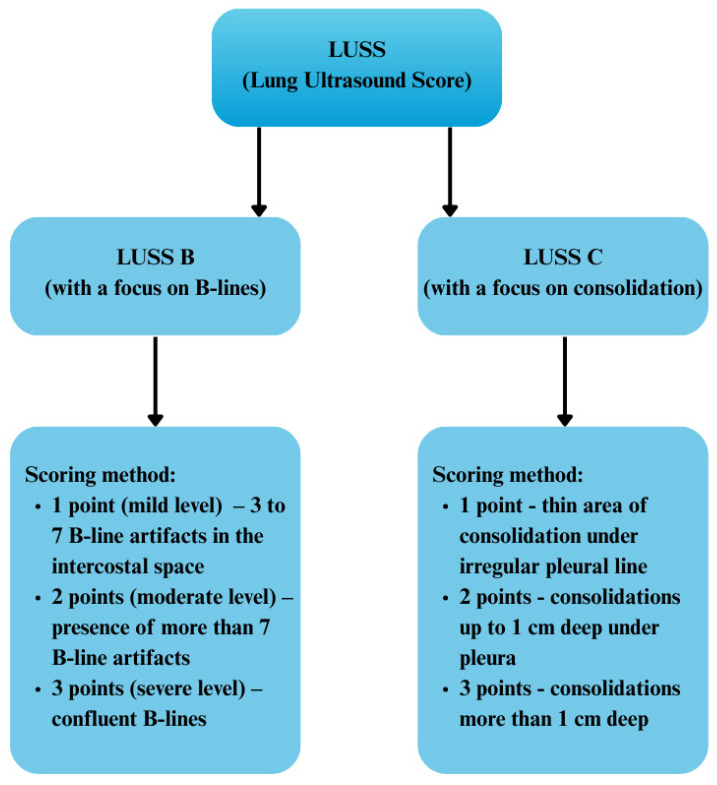
Ultrasound indicators reflecting the quantitative severity of inflammatory changes within the examined field.

Confirmation of the correlation between the pulmonary changes identified by lung ultrasound (LUS) and low-dose chest computed tomography (LDCT) was facilitated by implementing the ultrasound severity indices (LUSS B and LUSS C) that accounted for the quantitative extent of inflammatory changes within the examined lung fields. The detailed characteristics of these indices are presented in Figure 1.

The ultrasound scoring indices assessing the pulmonary inflammation without incorporating this modification did not validate the results presented below. Additionally, the maximum values for both of the scoring parameters were defined as follows:

• LUSS B max—no differentiation among severity grades 1, 2, or 3.
• LUSS C max—elimination of grade 1, with analysis restricted solely to grades 2 and 3.
• The LDCT protocol was required to meet the minimum criteria defined by the NCCN guidelines:
➢ Radiation dose for individuals with a BMI ≤ 30 did not exceed 3 mSv
➢ X-ray tube voltage: 100–120 kVp
➢ X-ray tube current: ≤40 mAs
➢ Detector collimation: ≤1.5 mm
➢ Reconstruction slice thickness: ≤1 mm
➢ Acquisition time: ≤15 s
• The scanning range of the LDCT examination extended from the lung apices to the costophrenic angles. The imaging was performed during deep inspiration, without the administration of intravenous or oral contrast agents. In cases where breath-holding was not feasible, scanning was conducted during shallow, quiet breathing.

To evaluate the pulmonary parenchymal involvement on the LDCT scans, the scoring system depicted in Table 1 was applied across each of the five lobes in both lungs.

**Figure 2:**
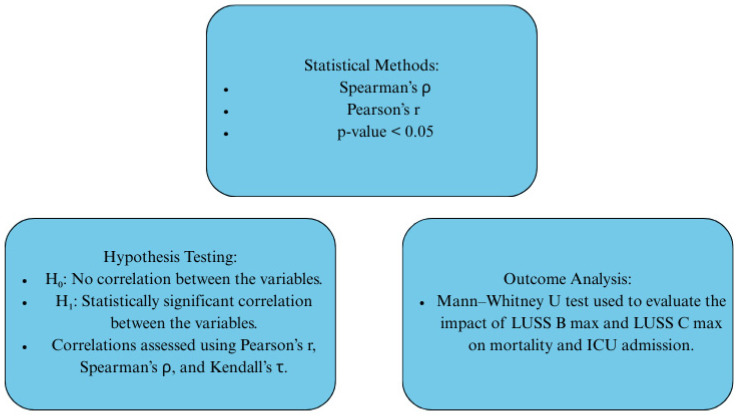
Data acquisition methods.

The statistical methods employed for data acquisition are outlined in Figure 2.

The severity of the B-line artifacts and pulmonary consolidations observed on the ultrasound displayed a linear correlation with the LDCT changes. The CTSS estimations derived from the lung ultrasound imaging parameters were calculated using the equations presented in Table 2.

Based on the previously described formulas, scatter plots were constructed and fitted with linear regression lines to illustrate the relationships between the CTSS and both the LUSS B and LUSS C scores.

**Figure 3:**
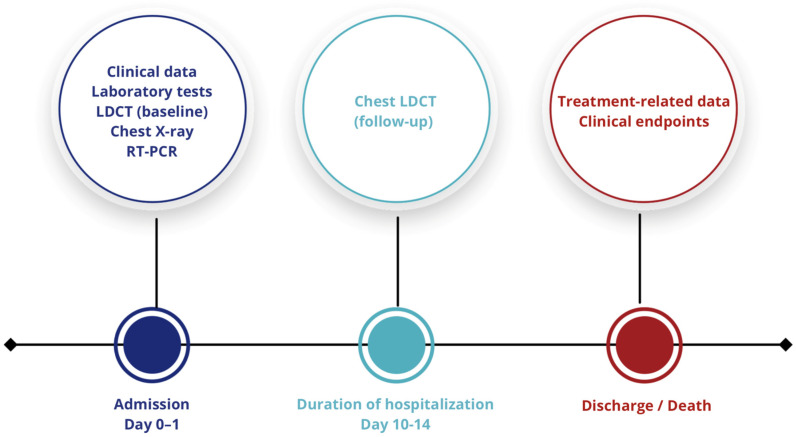
Study protocol flowchart. LDCT—low-dose computed tomography; RT-PCR—reverse transcription polymerase chain reaction.

Initially, a cohort of 1000 patients was screened for eligibility. Subsequently, applying predefined inclusion criteria and established time points, the study population was narrowed to 555 adult patients aged 18 years and older, as depicted in Figure 3.

Patients were not included in the study if they did not fulfill the inclusion criteria, particularly when follow-up chest CT scans (between days 10–14) were unavailable, laboratory or clinical data were incomplete, or treatment outcomes were uncertain, such as a transfer to another facility, discharge against medical advice, or death unrelated to COVID-19. No specific exclusion criteria related to comorbidities were outlined in the study protocol.

**Figure 4:**
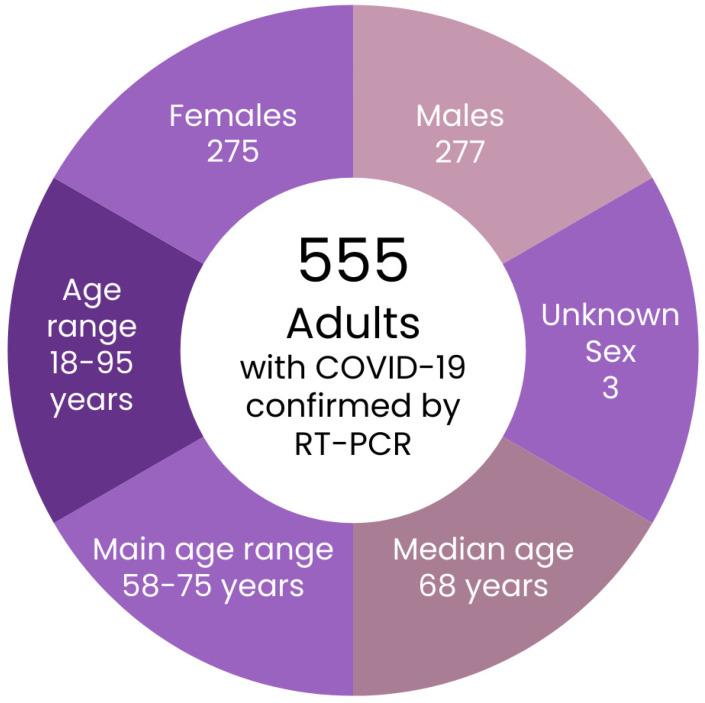
Characteristics of the study population. RT-PCR—reverse transcription polymerase chain reaction.

Importantly, LDCT findings were evaluated by a radiologist and supported by artificial intelligence algorithms. In patients presenting with auscultatory abnormalities, a statistically significant increase in ultrasonographic changes was observed. This relationship demonstrated a linear correlation: the severity of the ultrasound (US) findings approximately doubled the frequency of auscultatory phenomena, which were primarily associated with the presence of pulmonary consolidations. Based on these findings, a lung ultrasound was performed on a subgroup of 170 patients, and follow-up imaging was repeated after one week in 110 of these individuals, concurrently with an LDCT assessment. Detailed demographic and clinical characteristics of this population are presented in Figure 4.

Statistical analyses and 3D surface plots were performed using Statistica software, version 13.3 (TIBCO Software Inc., Palo Alto, CA, USA).

This research was carried out in adherence to the standards and guidelines outlined in the Frascati Manual, between 27 October 2020 and 26 January 2022, and was financially supported by the National Centre for Research and Development.

Of the literature cited, 97% was published within the past five years, underscoring the innovative character of this research field, as well as providing up-to-date information, recent findings, and unequivocally highlighting the topical relevance of the analyzed subject.

## 3. Results

The correlation between the pulmonary involvement severity index assessed by low-dose computed tomography (CTSS) and the quantitative severity indices derived from the lung ultrasound—specifically, LUSS C max (maximum consolidation severity score) and LUSS B max (maximum B-line severity score)—is presented in Table 3.

The impact of the LUSS B max (maximum B-line severity score) and LUSS C max (maximum consolidation severity score) on mortality and the necessity for intensive care unit (ICU) hospitalization, assessed using Mann–Whitney U test results, is presented in Table 4.

Subsequently, hypotheses H0 and H1 were tested by calculating Pearson’s, Spearman’s, and Kendall’s correlation coefficients. The obtained results are presented in Table 5.

**Figure 5:**
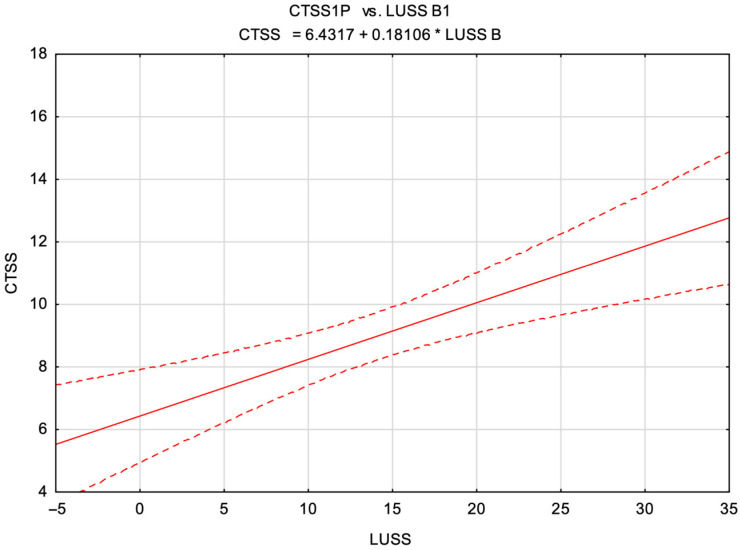
Linear regression between the CTSS and LUSS B. CTSS—chest CT severity score; LUSS B—lung ultrasound severity score with a focus on the B-lines.

Subsequently, a graph illustrating the relationship between the CTSS values and LUSS B scores was constructed (Figure 5).

**Figure 6:**
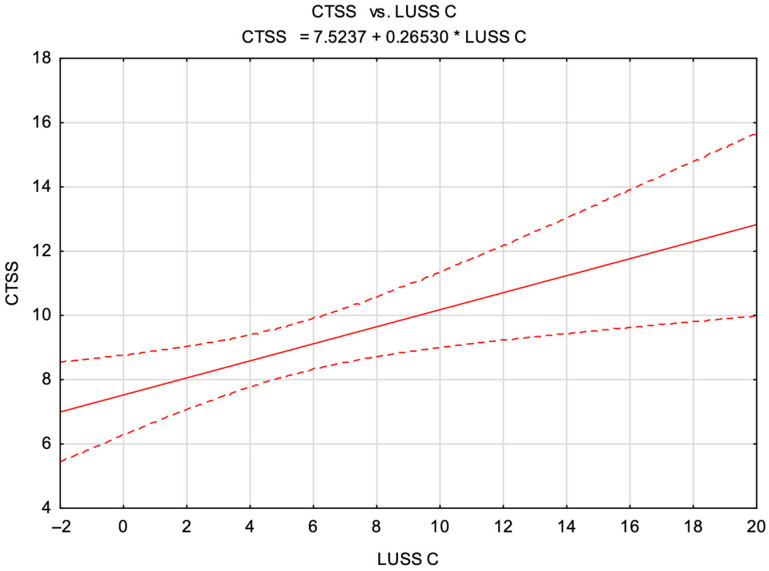
Linear regression between the CTSS and LUSS C. CTSS—chest CT severity score; LUSS C—lung ultrasound severity score with a focus on consolidation.

Analogously, a graph depicting the correlation between the CTSS values and LUSS C scores was generated (Figure 6).

**Figure 7:**
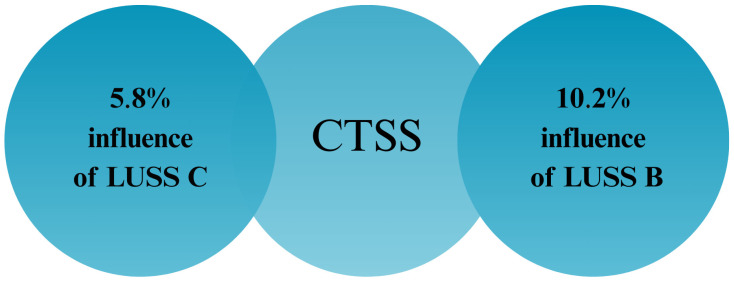
Percentage impact of the LUSS B and LUSS C on the CTSSs. CTSS—chest CT severity score; LUSS B—lung ultrasound severity score with a focus on the B-lines; LUSS C—lung ultrasound severity score with a focus on consolidation.

Finally, the percentage impact of LUSS B and LUSS C scores on the CTSS value was calculated and presented (Figure 7).

## 4. Discussion

The analysis of our findings confirmed that both low-dose computed tomography (LDCT) and lung ultrasound (LUS) are highly effective in identifying inflammatory lung alterations. Changes evaluated via the CT severity scoring system (CTSS) strongly correlated with the intensity of ultrasonographic artifacts, notably the B-lines and consolidations. Pulmonary consolidations, indicative of advanced COVID-19 disease and progressive inflammatory involvement [5], were detected by both imaging techniques, especially among patients with abnormal auscultation findings. Such concordance between the LUSS and CTSS has previously been documented in the literature, validating the consistency of these imaging modalities for evaluating pulmonary inflammatory involvement [6]. Existing meta-analyses similarly highlight the comparable diagnostic value of the LUSS and CTSS, though computed tomography generally offers superior sensitivity for detecting deeper and subpleural pulmonary lesions [7]. In our research, utilizing a semi-quantitative LUSS scale enabled robust comparisons between superficial consolidations and interstitial ultrasound artifacts with corresponding CTSS findings from LDCT imaging. However, in everyday clinical practice, the question arises: is LDCT accessibility always sufficient for the timely and repetitive monitoring of inflammatory dynamics?

Importantly, lesions detected by CT imaging were also appreciable by ultrasound, underscoring the considerable sensitivity of the LUS in identifying COVID-19-associated changes [8]. Nevertheless, the absence of standardized LUSS protocols—including defined evaluation zones and criteria for artifact interpretation—remains a substantial barrier for reliable comparisons between ultrasound and CT modalities [9]. In the present study, the application of a standardized semi-quantitative LUSS protocol proved effective, facilitating a quantitative ultrasound assessment and reliable correlation with CTSS findings. Such standardization notably enhanced the practical utility of ultrasound imaging for dynamic patient monitoring.

Notably, the adoption of a semi-quantitative LUSS system, permitting the precise evaluation of inflammatory severity, was pivotal. Only through standardized approaches did the correlation between the LUSS and CTSS become clear. Among patients with confirmed SARS-CoV-2 pneumonia, the LUSS findings have demonstrated predictive value regarding disease severity, correlating with CT findings and clinical parameters [10]. Traditional ultrasound assessments lacking semi-quantitative standardization failed to reveal such relationships, highlighting the necessity for uniform image analysis methodologies. Moreover, in our study, the lung ultrasound did not demonstrate the prognostic value in terms of ICU admissions or mortality. A possible explanation for this finding is the limited ability of the LUS to assess deeper parenchymal changes within the lungs, which may be important in predicting disease severity. Therefore, further studies correlating the LUS findings with CT imaging and clinical parameters are warranted to fully evaluate its usefulness in prognostication. Indeed, the literature underscores that the standardization of LUS protocols—including the number of evaluated fields, artifact classification, and scoring systems—is essential for achieving objective and reproducible inflammatory assessments, thereby enabling valid comparisons with CT [11]. Similar outcomes have been reported by other authors, demonstrating strong agreement between semi-quantitative LUSS and CTSS evaluations [8,12].

Furthermore, our results emphasize that lung ultrasound, despite its inherent limitations, frequently enables the rapid identification of clinically significant pathologies, such as consolidations or marked interstitial edema, which clinically manifest as dyspnea and respiratory insufficiency. Our observations align closely with previous studies describing LUSS as a frontline clinical assessment tool for COVID-19 patients [13,14].

Clinical observations further confirmed concordance between the LUS and LDCT in monitoring the dynamic progression of pulmonary inflammation. Follow-up examinations conducted after one week showed regression of lesions with both modalities: the ultrasound revealed reduced B-line artifacts and decreased consolidation extent, while LDCT demonstrated a corresponding decline in the CTSSs. The literature indicates that the simultaneous application of semi-quantitative scoring systems supports not only disease progression tracking but also assessment of therapeutic efficacy [15]. The utility of the LUSS as a supportive tool for intensive patient monitoring, particularly in intensive care settings, is also emphasized [16]. Furthermore, the severity levels assessed by computed tomography correlate closely with clinical symptoms, reinforcing CT’s role beyond diagnosis to prognostication [17]. Integrating imaging data with clinical parameters, including LDCT results, may significantly enhance decision-making processes regarding the COVID-19 severity assessment and hospitalization necessity [18].

On the other hand, despite agreement on the inflammation visualization, the lung ultrasound did not demonstrate prognostic utility regarding severe outcomes such as ICU admission or mortality risk. Ultrasound-derived scores failed to correlate significantly with these clinical parameters, thus limiting their prognostic value. These findings partially contrast with other studies linking LUSS scores to disease progression risks; discrepancies may arise from differences in applied protocols, operator experience, and patient demographics [19].

Nevertheless, it is crucial to underscore ultrasound’s role in clinical decision-making, particularly under healthcare system overload conditions. Lung ultrasound offers undeniable practical advantages, being rapid, accessible, safe, and repeatable at the bedside without exposing patients to ionizing radiation. Unlike LDCT, whose availability is frequently limited during healthcare crises, lung ultrasound may be performed using portable devices even in hemodynamically unstable patients, pediatric populations, and pregnant women, rendering it a viable alternative for daily clinical practice [20,21].

Additionally, advances in artificial intelligence (AI) technology in COVID-19 imaging deserve a special mention. This study utilized AI algorithms for the quantitative analysis of CT images, improving objectivity, enhancing measurement reproducibility, and minimizing the human factor influence. Parallel approaches reported in the literature underscore AI’s potential in image classification and diagnostic streamlining [22,23,24,25].

In summary, lung ultrasound and low-dose computed tomography should not be viewed as competing modalities but as complementary tools for the diagnosis and monitoring of COVID-19 patients. Whereas LDCT provides high-resolution, precise delineation of the pulmonary inflammatory extent and characteristics, lung ultrasound excels in mobility, safety, and bedside repeatability, rendering it particularly valuable amid dynamic clinical conditions. Integrated use of both imaging techniques, particularly when coupled with standardized scoring systems (such as the CTSS and LUSS), substantially enhances the therapeutic decision-making accuracy and patient care quality, especially in resource-limited healthcare settings such as long-term and primary care [26,27].

Our findings—including significant correlations between LUSSs and CTSSs and observed lesion dynamics on follow-up imaging—reinforce the utility of the lung ultrasound for diagnosis and monitoring of SARS-CoV-2-infected patients. Future studies should focus on validating the LUSS scale across diverse patient populations and developing AI-supported algorithms for objective ultrasound image analysis.

In the context of the COVID-19 pandemic, comprehensive integration of lung ultrasound, low-dose CT imaging, and artificial intelligence technologies represents a critical diagnostic strategy, facilitating effective patient management even amid significant healthcare system strains.

Nonetheless, this study has several limitations requiring consideration. First, lung ultrasound inherently possesses limited specificity—artefacts such as the B-lines and consolidations may also occur in other pathologies such as pulmonary fibrosis or heart failure. Consequently, the LUSS findings must be interpreted strictly within clinical and laboratory contexts.

Additionally, subjective ultrasound assessments without standardized scoring showed no significant correlation with the CT severity. Only objective, numeric-based evaluations provided comparable results. Existing literature indicates the predictive quality of the LUSS depends greatly on operator expertise and applied protocols [28]. Lack of appropriate training and standardization procedures in lung ultrasound significantly diminishes result reliability and interpretation accuracy [29]. According to ACEP guidelines for point-of-care ultrasound, the accuracy of the LUSS strongly depends on the operator’s experience and strict adherence to scanning protocols [30].

Another limitation is the absence of study randomization and the lack of result validation in external populations, restricting the generalizability of our observations.

It is important to emphasize that in the present study, the group of radiologists involved in image interpretation was relatively small. Consequently, inter-reader agreement metrics, such as kappa statistics, were not calculated.

Finally, our analysis underscores that only standardized, quantitative ultrasound evaluation yields results comparable with computed tomography, emphasizing the ongoing need for the further standardization of ultrasound imaging assessment methods. Careful, continued standardization efforts are indispensable for ensuring the optimal clinical utility of ultrasound in future healthcare scenarios, particularly when combined with patients’ clinical outcome data.

## 5. Conclusions

Our findings demonstrate a statistically significant, moderate positive correlation between lung ultrasound severity scores—specifically LUSS B and LUSS C—and chest CT severity scores (CTSSs), affirming the clinical utility of the lung ultrasound as a supportive imaging tool for evaluating the inflammatory severity in COVID-19 pneumonia. Incremental increases of 1 point in LUSS B and LUSS C correspond to mean increases in the CTSS values of approximately 0.18 and 0.27 points, respectively. Despite the statistical significance, the observed relationships remain moderate, emphasizing that lung ultrasound represents a valuable adjunct, rather than a complete substitute for LDCT-based pulmonary assessment.

Both imaging modalities successfully facilitated the quantitative analysis of inflammatory dynamics, with the standardized LUSS protocol notably enhancing the accuracy and reproducibility of ultrasonographic assessments. Although low-dose computed tomography exhibited superior predictive accuracy regarding severe disease progression, lung ultrasound emerges as a pragmatic and highly beneficial diagnostic alternative, particularly in resource-limited healthcare environments lacking immediate access to advanced imaging technologies.

Crucially, our results highlight an urgent need for the ongoing standardization of lung ultrasound protocols and emphasize the promising potential of integrating ultrasound imaging with advanced artificial intelligence algorithms. Future research should prioritize developing and validating AI-supported LUS systems based on multicenter data, thereby substantially increasing the clinical relevance, diagnostic precision, and prognostic capabilities of lung ultrasound in managing patients with COVID-19. Such efforts aim to establish robust, generalizable tools that can assist clinicians in timely and accurate decision-making across diverse healthcare settings.

## Figures and Tables

**Table 1:** Description of the CTSS scoring system. CTSS—chest CT severity score.

CTSS	Extent of Lobe Involvement
1 point	<5%
2 points	5–25%
3 points	26–50%
4 points	51–75%
5 points	>75%

**Table 2:** Mathematical formulas enabling the estimation of the CTSSs. LUSS B—lung ultrasound severity score focusing on the B-lines; LUSS C—lung ultrasound severity score focusing on consolidations; CTSS—chest CT severity score; F—statistical test; *p*—significance level.

	Formula
LUSS B	CTSS = 0.18(±0.05) × LUSS B + 6.43(±0.75) ± 4.38 (F = 14.6, *p* = 0.0002)
LUSS C	$CTSS=0.27\left(\pm 0.09\right)\times LUSS\;C+7.25(\pm 0.63)\pm 4.48$ (F = 7.9, *p* = 0.006)

**Table 3:** Correlation between the CTSS and the LUSS B max or LUSS C max. CTSS—chest CT severity score; LUSS C max—maximum consolidation severity score from the lung ultrasound; LUSS B max—maximum B-line severity score from the lung ultrasound.

Correlation	Spearman’s Correlation Coefficient (r)	Spearman’s *p*-Value	Pearson’s Correlation Coefficient (r)	Pearson’s *p*-Value	Statistical Significance
CTSS vs. LUSS C max	0.2	0.03	0.18	0.04	Yes
CTSS vs. LUSS B max	0.1	0.24	0.12	0.17	No

**Table 4:** Impact of the LUSS B max and the LUSS C max on mortality and ICU admission. LUSS C max—maximum consolidation severity score from the lung ultrasound; LUSS B max—maximum B-line severity score from the lung ultrasound; *p*—significance; ICU—intensive care unit.

	*p*—for Death	*p*—for ICU
LUSS C max	0.185	0.895
LUSS B max	0.351	0.638

**Table 5:** Correlation between the CTSS and the LUSS B or LUSS C. CTSS—chest CT severity score; LUSS B—lung ultrasound severity score with a focus on the B-lines; LUSS C—lung ultrasound severity score with a focus on consolidation.

Correlation	Pearson’s Correlation Coefficient (r)	Pearson’s *p*-Value	Spearman’s Correlation Coefficient (r)	Spearman’s *p*-Value	Kendall’s Correlation Coefficient (τ)	Kendall’s *p*-Value
CTSS vs. LUSS B	0.32	0	0.33	0	0.25	0
CTSS vs. LUSS C	0.24	0.006	0.227	0.003	0.197	0.002

## Data Availability

Any data will be provided by the authors if requested.
